# Identification and characterization of a novel *TACSTD2* mutation in gelatinous drop-like corneal dystrophy

**Published:** 2010-04-28

**Authors:** Preeti Paliwal, Jaya Gupta, Radhika Tandon, Namrata Sharma, Jeewan S. Titiyal, Seema Kashyap, Seema Sen, Punit Kaur, Divya Dube, Arundhati Sharma, Rasik B. Vajpayee

**Affiliations:** 1 Laboratory of Cyto-Molecular Genetics, Department of Anatomy, All India Institute of Medical Sciences, New Delhi, India; 2 Dr. Rajendra Prasad Centre for Ophthalmic Sciences, All India Institute of Medical Sciences, New Delhi, India; 3 Department of Biophysics, All India Institute of Medical Sciences, New Delhi, India; 4 Centre for Eye Research Australia, University of Melbourne, Australia

## Abstract

**Purpose:**

To study the clinical, histological, in vivo confocal microscopic, and molecular profile in a family with gelatinous drop-like corneal dystrophy (GDLD) from north India.

**Methods:**

Two siblings from a consanguineous family presented with clinical features analogous to GDLD. Detailed clinical evaluations were performed for all the available affected and unaffected members of this family. In vivo confocal microscopy and histology was done wherever necessary. DNA isolated from peripheral blood samples was subjected to polymerase chain reaction (PCR) followed by direct sequencing to detect mutations in the tumor-associated calcium signal transducer 2 (*TACSTD2*) gene. Protein modeling studies were done to asses the effect of the mutation on the protein structure.

**Results:**

The diagnosis of GDLD was established in the patient and the affected sibling on slit-lamp examinations, which revealed mulberry-like opacities in the subepithelium and anterior stroma that were confirmed on histopathology. The findings of the in vivo confocal microscopy were consistent with those reported in previous reports. Sequencing *TACSTD2* revealed a novel homozygous missense mutation c.356G>A, leading to amino acid substitution C119Y in the two affected siblings. The mutation was found to be pathogenic on Sorting Intolerant From Tolerant (SIFT) analysis and was not found in normal controls and unaffected individuals of the family. A synonymous, previously reported, single nucleotide polymorphism (SNP; rs13267) was also seen in all the individuals of the family. Protein modeling studies involving wild-type and mutant protein indicated an exposed cysteine residue in the mutant protein.

**Conclusions:**

A novel *TACSTD2* C119Y mutation leading to an amino acid substitution was identified in two affected siblings of a family. Protein modeling studies revealed an exposed cysteine residue, which might cause interchain disulfide bond formation and protein aggregation leading to disturbed cell junctions of the corneal epithelium.

## Introduction

Gelatinous drop-like corneal dystrophy (GDLD; OMIM 204870), first described by Nakaizumi [1], is a rare type of primary amyloidosis, with highest prevalence reported from Japan (1 in 30,000). Clinically, the dystrophy is characterized by an accumulation of yellowish-white, mulberry-like, gelatinous, amyloid masses in the subepithelial region of the cornea. The accumulation of these masses causes loss of vision, foreign-body sensation, photophobia, and lacrimation. GDLD usually appears in the first decade of life, and neovascularization of the subepithelial and superficial stroma is seen in the later stages. Patients often require multiple surgical interventions as the disease is reported to recur within a few years.

GDLD is an autosomal recessive disorder and is known to be caused from mutations in the tumor-associated calcium signal transducer 2 (*TACSTD2*) gene located on the short arm of chromosome 1 [1p32] [2]. Analysis of patients from different ethnic backgrounds shows the presence of genetic heterogeneity [3]. *TACSTD2* is 2.07 kb in length and has one exon encoding the tumor-associated antigen, which is a transmembrane glycoprotein of 323 amino acids. This protein is a monomeric cell surface glycoprotein expressed in the cornea, trophoblasts, and in most carcinomas [4,5]. Its function is largely unknown and is speculated to act as a cell–cell adhesion receptor in cancer cells and as a calcium signal transducer [5]. It has been speculated that the loss of function of the gene in corneal tissue results in an increased permeability of corneal epithelium, resulting in the GDLD phenotype. To date, 24 mutations (Table 1) have been identified in *TACSTD2* in patients affected with GDLD [5-7]. In the present study we aimed to identify the underlying mutations in *TACSTD2* in the family and for genotype–phenotype correlation. Protein homology modeling and molecular dynamics (MD) simulations were performed to assess the structural implications of the underlying mutation on the protein conformation.

**Table 1:** *TACSTD2* mutations reported in various studies.

**Study number**	**Mutation**	**Amino acid change**	**Reference**
1	c.2T>G	Met1Arg	[6]
2	c.84insG	28fsThrXGlu93X	[7]
3	c.198C>A	Cys66X	[6]
4	c.250A>T	Lys84X	[6]
5	c.322T>C	Cys108Arg	[6]
6	c.341T>G	Phe114Cys	[7]
7	c.352C>T	Gln118X	[6]
8	c.352C>G	Gln118Glu	[6]
9	c.355T>A	Cys119Ser	[6]
10	c.480delC	His160fs174GluX	[7]
11	c.493_494insCCACCGCC	Gly165AlafsX15	[6]
12	c.509C>A	Ser170X	[6]
13	c.519dupC	Ala174ArgfsX43	[6]
14	c.526_576del51	del.176_192	[7]
15	c.551A>G p	Tyr184Cys	[6]
16	c.557T>C	Leu186Pro	[6]
17	c.564delC	Lys189SerfsX82	[6]
18	c.581T>A	Val194Glu	[6]
19	c.619C>T	Gln207X22	[6]
20	c. 632delA	Gln211ArgfsX60	[6]
21	c.653delA	Asp218ValfsX53	[6]
22	c.679G>A	Glu227Lys	[7]
23	c.772_783delATCTATTACCTGinsT	lle258X 772 to 783del	[6]
24	c.811delA	Lys271SerfsX26	[6]

## Methods

### Patients and control subjects

The study adhered to the tenets and declaration of Helsinki, and the protocol was approved by the Institutional Ethics Committee of the All India Institute of Medical Sciences (AIIMS). The patients, an 18-year-old male and his 10-year-old sister, presented at the Cornea Clinic of Dr. Rajendra Prasad Center for Ophthalmic Sciences, All India Institute of Medical Sciences (New Delhi, India) with diminution of vision, photophobia, and lacrimation when he was 6 years old and his sister was 8 years old.

**Figure 1:**
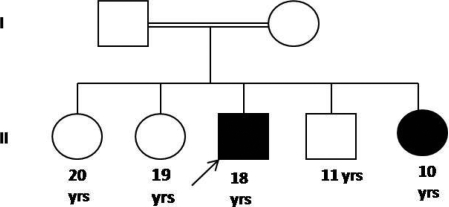
Pedigree of the family with gelatinous drop like dystrophy (GDLD). Filled boxes represent individuals affected with GDLD. Open boxes represent unaffected individuals. Arrowhead indicates the proband. A double line indicates presence of consanguinity in the family. The numerals shown below the symbols indicate the age of the individuals.

Detailed pedigree information was collected. All the available family members—their parents and one unaffected sibling—were clinically examined (Figure 1). Diagnosis of GDLD was based on detailed clinical evaluation that included slit-lamp examination, in vivo white-light confocal microscopy, specular microscopy, pachymetry, orbscan, and ultrasound for posterior segment evaluation.

A total of 50 healthy volunteers with no ocular or other disorders matched for age (range between 8-20 years) and sex (25 males and 25 females) formed the controls. From both the patients and controls, 5 ml of peripheral venous blood sample was collected in EDTA after taking informed consent and explaining the nature and possible consequences of study participation. These samples were processed immediately for DNA extraction.

### Histopathology

The brother underwent surgical intervention, and histopathological evaluations were performed on the corneal tissue sample. It was sectioned and analyzed with light microscopy after staining with hematoxylin and eosin and Congo red stains.

### In vivo confocal microscopy

In vivo confocal microscopy was performed using ConfoScan 4.0 (Nidek, Inc., Freemont, CA) attached to an immersion lens (Achroplan 40X/0.75 W). The central cornea was examined after instilling a drop of 0.5% proxy metacine hydrochloride (Paracaine, Sunways, Mumbai, India). Gen Teal gel (Novartis India Ltd, Hyderabad, India) was used as a coupling medium to cover the front surface of the immersion lens. The images had an axial resolution of 10 µm.

### Mutation analysis

Genomic DNA was extracted from peripheral blood samples using a DNeasy kit (Qiagen, GmbH, Hilden, Germany) The DNA was amplified using the primer pairs described previously (Table 2) [6].The cycling conditions were 15 min denaturation at 95 °C, followed by 35 cycles of 94 °C for 45 s, 58 °C for 30 s, 72 °C for 1 min 30 s, and a final extension at 72 °C for 5 min. The reaction mixture of 25 μl was prepared using 200 ng genomic DNA, primers (0.5 µM each), MgCl_2_ (1.5 mM), deoxyribonucleotide triphosphates (dNTPs; 0.2 mM), 1× PCR buffer (containing 10 mM TRIS-HCl, pH 8.3, 50 mM KCl; Roche, Applied Biosystems, Foster City CA), and Taq polymerase (1 U; Roche, Applied Biosystems).

**Table 2:** GDLD PCR primer sequences.

**Primer name**	**Direction**	**Primer sequence**
M1S1-Fa	Forward	5'-GAGTATAAGAGCCGGAGGGAG-3'
M1S1-Ra	Reverse	5'-CATCGCCGATATCCACGTCAC-3'
M1S1-Fb	Forward	5'-CTGAGCCTACGCTGCGATGAG-3'
M1S1-Rb	Reverse	5'-GGATCTATTAAACCTGGTGTGTG-3'

The amplified products of patients and controls were sequenced bidirectionallly after purification with Qiaquick kit (Qiagen) with BigDye Terminator Mix version 3.1 (Applied Biosystems), and were then analyzed on an ABI-3100 Genetic Analyzer (ABI). Nucleotide sequences for the coding regions were compared with the nucleotide sequence of the published *TACSTD2* human cDNA (ENST00000371225).

### In silico analysis

This was done using the Sorting Intolerant From Tolerant (SIFT) tool, which generates multiple alignments of the sequence over different species to look at the conserved sequences of a gene; it assesses the conserved amino acid positions and analyzes the effect of missense changes on the conserved structure of proteins over the course of evolution. The SIFT tool assigns a score to the mutations, and the score of <0.05 is considered potentially damaging.

### Homology modeling of mutant protein

A search through the Protein Data Bank revealed that the structural template was available only for the thyroglobulin type 1A (TY) domain of the TACSTD2 protein. The TY domain of TACSTD2 in the present case is the domain that harbors the single C119Y mutation. In an attempt to examine the structural implications of this mutation, homology modeling and MD simulations were performed using the template of the TY domain. The model of the native protein was built involving the stretch from 89 to 145 amino acid residues by following a fold prediction protocol with the help of the Phyre server [8]. The homology model of the native domain was built using the three-dimensional X-ray crystal structure of major histocompatibilty complex (MHC) class II-associated P41 II fragment (PDB code: 1ICF) as a template with the protein modeling module of Discovery Studio 2.0 (Messrs; Accelrys, San Diego, CA). Mutant (C119Y) was generated by altering the corresponding residue, and the resulting native and mutated structures were minimized separately employing MD simulation. The sequence analysis of the TY repeat of TACSTD2 (wild type) showed that the protein contains six conserved cysteine residues, five of which could be modeled from this structural template.

## Results

### Clinical findings

**Figure 2:**
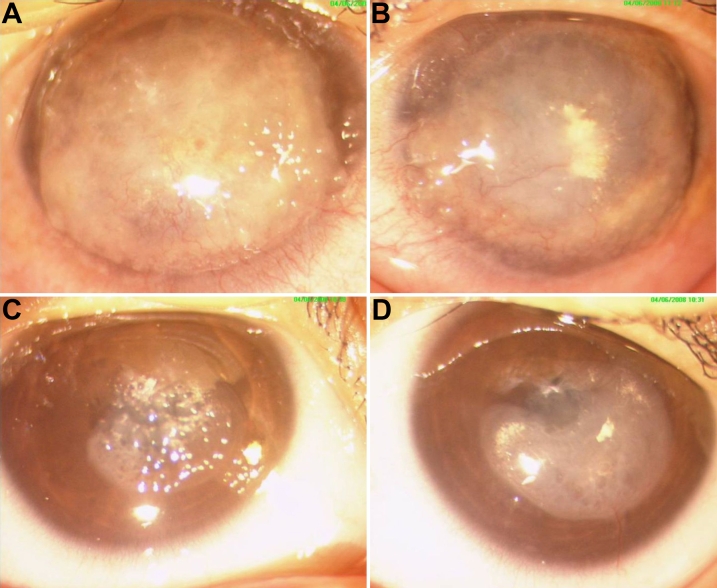
Clinical photomicrographs of the affected individuals of the family. The right eye (**A**) and left eye (**B**) show advanced paracentral mulberry like deposition with nodular opacification and neovascularization in the affected brother.  Right eye (**C**) of the affected sibling shows typical white sub-epithelial nodules with mulberry pattern in the central cornea and the left eye (**D**) of the same affected sibling shows band like opacity in the interpalpebral area with neovascularization.

Slit-lamp examination of the 18-year-old patient with GDLD revealed bilateral, advanced, mulberry-like, elevated, whitish, nodular, vascularized opacities in the subepithelial region (Figure 2A,B). He had previously undergone penetrating keratoplasty in the right eye at our hospital in the year 2000 and a lamellar keratoplasty of the left eye in 2002. In the year 2008 he presented with recurrence of dystrophies in both eyes. He again underwent deep anterior lamellar keratoplasty in the left eye. Slit-lamp examination at 1-year follow-up revealed the best corrected visual acuity for the left eye as 20/80.

The affected younger sister, who was examined at 10 years of age, had photophobia and lacrimation in both eyes for the past 2 years. Her BCVA was 20/80 in the right eye and 20/60 in the left eye. Slit-lamp examination revealed band-shaped corneal opacities in the interpalpebral area of the cornea of both eyes, with several gelatinous prominences above the band-shaped opacity in the right eye (Figure 2C). There was vascular invasion from the inferior limbus into the clear cornea in the left eye (Figure 2D). No surgical intervention had been advised as the patient was not visually handicapped and was comfortable with tear substitutes.

Slit-lamp examinations established the diagnosis of GDLD in the two siblings. Clinical evaluation of the parents and the unaffected sibling did not reveal any ocular abnormalities.

### In vivo confocal microscopy

**Figure 3:**
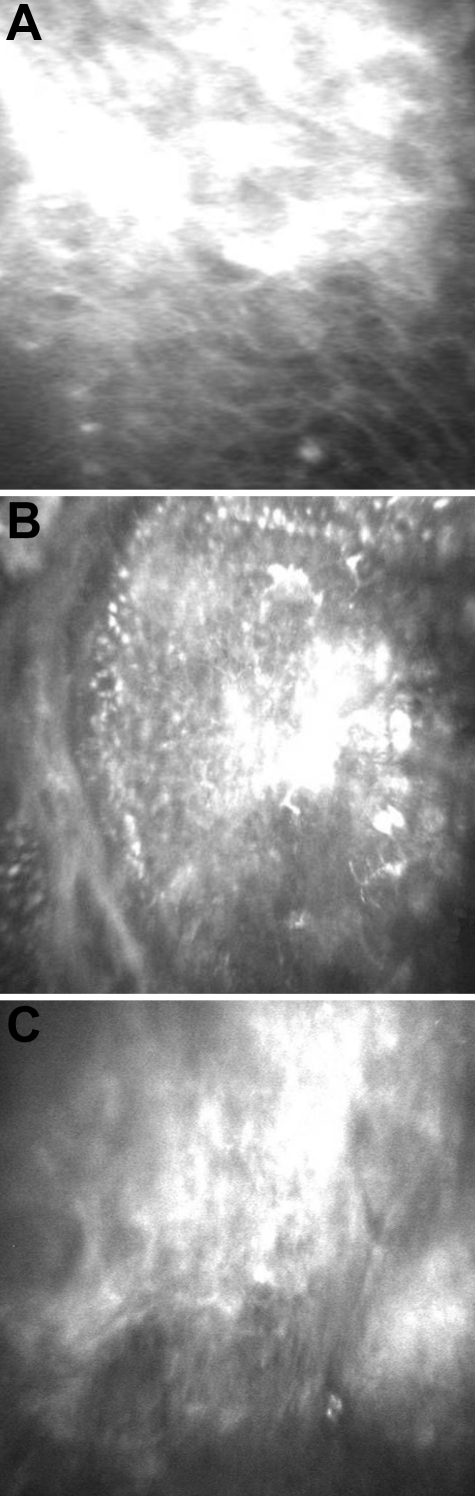
In vivo confocal microscopic findings of the affected individual. **A**: Superficial epithelial cells and **B**: basal epithelial cells with a well-demarcated cell border and the presence of reflective material are seen. **C**: Stroma/endothelium is not apparent.

In vivo confocal microscopy of the affected brother revealed homogenous reflective material at the basal epithelium with well demarcated cell borders. The Bowman’s layer was replaced with this reflective material. Only the superficial epithelium was apparent, while sub-basal nerve plexus, stroma, and endothelium could not be visualized (Figure 3).

**Figure 4:**
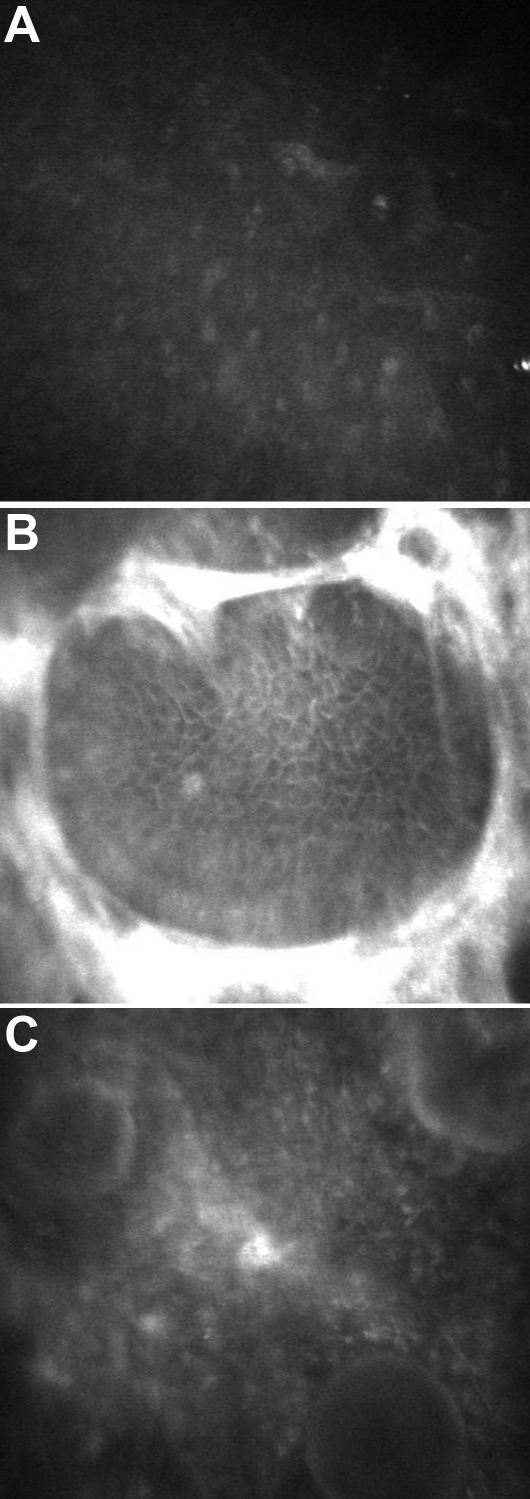
In vivo confocal microscopic findings of the affected sibling. Normal superficial epithelium is shown (**A**). Basal epithelim with circumscribed hyperreflectivity is seen (**B**). Anterior stroma shows globular drop like structures (**C**).

In vivo confocal microscopy of the affected sister revealed a normal superficial epithelium with homogenous reflective material at the basal epithelium with well demarcated cell borders. In addition, circumscribed hyper-reflectivity and many drop-like globular structures were seen in the left eye, manifesting with a band-like opacity (Figure 4). The Bowman’s membrane was replaced with the reflective material, the sub-basal nerve plexus was not apparent in either eye; however, the underlying stroma, keratocytes, and endothelium were normal.

### Histological findings

**Figure 5:**
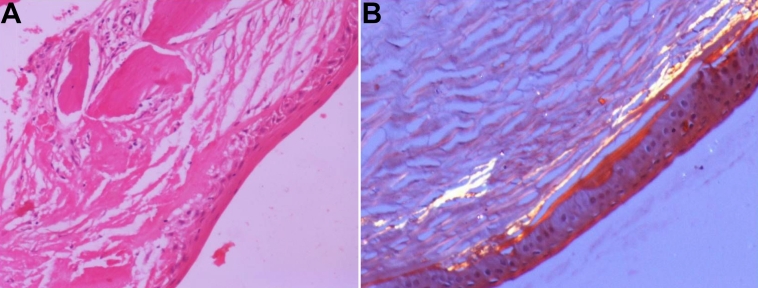
Histopathological findings. **A**: Hematoxylin and eosin staining at 20× reveals atrophied overlying epithelium and Bowman’s membrane. The subepithelium and superficial stroma are seen containing eosinophilic material. **B**: Amyloid deposition was confirmed in the anterior and posterior stroma as birefringence was seen on viewing under a polarized filter.

Histopathology confirmed the presence of amyloid in the cornea on staining with Congo Red. Epithelial atrophy was observed. The anterior stroma showed acellular eosinophilic deposits in the subepithelium and superficial stroma along its entire length. All the Congo red-positive deposits of amyloid exhibited apple-green birefringence under polarized light (Figure 5).

### Mutation analysis

**Figure 6:**
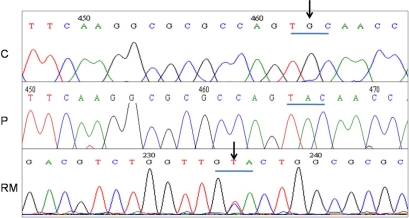
Sequencing results of the tumor associated calcium signal  transducer 2 (*TACSTD2*) gene in the family. Partial nucleotide sequence of the patient (P) shows the novel mutation c.356G>A (C119Y) in comparison to normal control sequence (C). The reverse complement of the partial nucleotide sequence of the mother (RM) shows the change in heterozygous state. Arrowheads indicate position of the mutation.

Direct sequencing revealed a novel mutation in *TACSTD2* in GDLD. The homozygous missense mutation C119Y (GenBank GU138371) was seen in both the affected individuals. The same change was not seen in the unaffected brother and 50 controls also sequenced. The change was present in the heterozygous state in the parents. A previously reported synonymous single nucleotide polymorphism (SNP; rs13267) was found in *TACSTD2* in all the family members (Figure 6).

### Sorting intolerant from tolerant (SIFT) analysis

In silico analysis showed that position 119 was conserved in all orthologues. The SIFT tool analysis revealed a score of <0.05 and predicted that the replaced amino acid is potentially damaging and would not be tolerated (Table 3).

**Table 3:** Multiple sequence alignment of *TACSTD2* from different species. The amino acid cysteine (C) at position 119 conserved in all the orthologs is highlighted in bold.

*Homo sapiens*	DGLYDPDCDP EGRFKARQCN QTSVCW**C**VNS VGVRRTDKGD LSLRCDELVR
*Mus musculus*	DGLYDPECDD KGRFKARQCN QTSVCW**C**VNS VGVRRTDKGD QSLRCDEVVR
*Macaca mulatta*	DGLYDPDCDE SGLFKAKQCN GTSTCW**C**VNT AGVRRTDK-D TEITCSERVR
*Bos Taurus*	DGLYDPECDD KGLFKAKQCN GTSTCW**C**VNT AGVRRTDK-D SEISCSEPVR
*Xenopus laevis*	DGLYDPECET NGVFKARQCN NTDTCW**C**VNT AGVRRTDKGD KNWKCPELVR
*Gallus gallus*	DGLYDPECEN NGLFKAKQCN GT-TCW**C**VNT AGVRRTDKHD TDLKCNQLVR
*Danio rerio*	DGIYDPECQS DGKFKAVQCN NTEVCW**C**VNS AGVRRSDKKD KNIKC-EPAE
*Trichoplax adhaerens*	GVYIPECNP DGSFAGLQCD STKYCW**C**VNI FGXXXXXXXX XXXXXXXXXX

### Protein modeling

**Figure 7:**
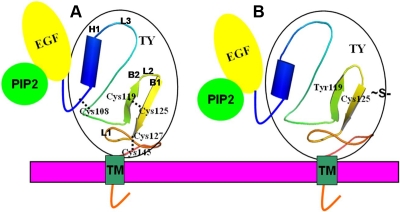
Schematic representation of both wild type (**A**) and mutant (**B**) tumor associated calcium signal transducer 2 (TACSTD2) protein. The modeled structure is encircled in black. The abbreviations used are: epidermal growth factor (EFG)-like repeat, a thyroglobulin type 1A (TY) repeat, a transmembrane domain (TM) and a phosphatidylinositol (PIP2)- binding site, L1, L2, L3 are loops, H1 is helix while B1 and B2 are anti-parallel b-sheets . The free cysteine residue in the mutant protein is depicted by ~S- and disulphide bridges by dashed lines.

**Figure 8:**
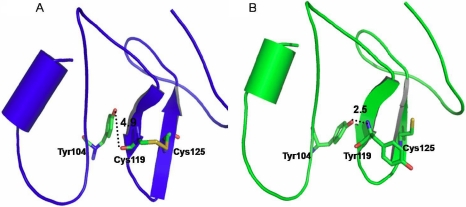
Comparison of the wild-type (**A**) shown in blue and mutant conformers (**B**) shown in green obtained after Molecular Dynamics (MD) simulations. Difference in distances of wild type (4.9Ǻ) and mutant (2.5 Ǻ) are indicated. The change in secondary structure element is due to formation of hydrogen bond interactions shown by dashed lines.

The secondary structure of the TY domain comprises an antiparallel β-sheet conformation (B1 and B2) and a helix (H1) joined by loops (L2 and L3). The disulphide bridge in the domain Cys119–Cys125 is present between the β-sheets, and Cys127–Cys145 stabilizes the loop conformation at the COOH terminal of the domain (Figure 7). A comparison of the MD conformers in both mutant C119Y and wild-type structures revealed a very subtle variation in the overall geometry of the protein with a root mean square deviation (rmsd) variation of 0.8 Å in the backbone atoms. The model quality was also checked by the ProsaII server [8,9] with a *Z* score of −2.81, which indicates that the score lies within the range of scores typically found for native proteins of similar size. The overall geometry of the protein is seen to be conserved even in the presence of the C119Y mutation (Figure 8).

## Discussion

Phenotypic variants are known for GDLD [10-12], and no clear genotype phenotype correlation has been established [13-15]. In vivo confocal microscopy can provide useful insight into the disease and thus serve as an important tool in characterizing and identifying GDLD. Only one report is available describing the in vivo confocal microscopy findings in GDLD [7]. The findings in our case are consistent with those mentioned in the earlier study.

To our knowledge, this is the first report of a novel mutation in patients with GDLD from north India. The C119Y mutation in *TACSTD2* co-segregated with the disease phenotype in the pedigree, whereas no mutations were found in the controls analyzed. The data show that the C119Y mutation in *TACSTD2* is responsible for GDLD in these patients.

To date, approximately 24 different mutations have been identified in *TACSTD2* (Table 1). Q118X is the most commonly reported mutation [6]. Previous reports have also suggested that amino acid substitution at position 119 is responsible for causation of GDLD [15]. Position 119 was seen to be conserved among the orthologues by in silico analysis. This part of the *TACSTD2* sequence is considered to encode a functionally important part of the protein [15]. The 40-kDa TACSTD2 protein contains an epidermal growth factor-like repeat, a thyroglobulin repeat, a transmembrane domain, and a phosphatidylinositol-binding site harboring phosphorylatable serine and threonine residues near the COOH terminus [2]. The mutation detected in this study affects the TY domain of the protein, resulting in loss of the cysteine residue at position 119, which is involved in disulfide bond formation. This TY domain is formed by 76 of the 323 amino acids of TACSTD2; loss of a disulfide bond does not alter the protein structure, and the overall geometry of the protein is conserved, even in the presence of the C119Y mutation. This indicates that the disulphide bridge may not be essential for the stability of the antiparallel β-sheet conformation as these are stabilized by hydrogen bonds [16]. On closer analysis, however, a deviation in the secondary structure element was observed. The mutation leads to the shortening of one of the β-sheets (B1) by two residues (from Cys125 to Ser123; Figure 7). The change in the secondary structure elements could be attributed to the formation of an additional hydrogen bond between Tyr104 and Tyr119 in the mutant C119Y protein. This mutation also leads to an extra free cysteine residue in the protein. The free cysteine residue (Cys125) in the mutant protein is exposed to the protein surface (Figure 8). The positioning of this free amino acid residue indicates that it could be involved in the formation of disulfide bridges with other similar mutated molecules having a free cysteine residue. Therefore, we postulate that the formation of molecular dimers as a result of the intermolecule disulphide bridge from this mutation is probably responsible for protein aggregation.

The TY domain contains the thyroglobulin type-1 repeat signature [17] with six conserved cysteines and is thought to be involved in the control of proteolytic degradation. Studies using transfection assays have shown normal TACSTD2 protein to accumulate at cell to cell contact borders [18]. It has also been shown that truncated proteins do not accumulate at the cell membrane [2] and thus cause disturbances in the cell to cell and cell to substrate adhesion function, which is evident by abnormal gaps between surface epithelial cells, disordered desmosomes, and abnormal basal epithelium [19]. Our protein modeling studies also show an excess of free cysteine involved in disulphide bond formation with similar mutated proteins and forming aggregates. We hypothesize that these aggregates of abnormal proteins accumulate in the cytoplasm and are unable to reach the cell membrane, thus altering the cell to cell adhesion function of the corneal epithelium. It has also been demonstrated that mutated TACSTD2 is associated with increased cell permeability compared to controls; this disturbs the cell to cell adhesion and contributes to pathogenesis of amyloid deposition  [19]. Lactoferrin, a protein of the lacrimal glands, has been identified in GDLD amyloid deposits [20] and is thought to originate from the tear film and penetrate the disturbed cell junctions [19] in the basement membrane and subepithelial area. These findings, along with the functional analysis of TACSTD2, may be critical in elucidating the pathogenesis of GDLD.

In conclusion, we report for the first time the clinical, in vivo microscopic, histologic, genetic, and protein modeling findings of a family with GDLD. A novel *TACSTD2* C119Y mutation was identified in two affected siblings of a family in whom in vivo confocal microscopy and histopathology results were consistent with clinical findings. In addition, characterization of the mutation using homology modeling suggested that the exposed cysteine in the mutant protein may cause interchain disulfide bond formation, leading to protein aggregates which are unable to reach to the cell boundaries and accumulate at the cell to cell adhesion borders, thereby disturbing the cell to cell junctions. This disturbance may lead to increased permeability of the corneal epithelial cells.
